# A Mixed Methods Approach to Understanding Curricular Impact of a Capstone Course on the Self-Efficacy of Fourth-Year Medical Students

**DOI:** 10.7759/cureus.9537

**Published:** 2020-08-03

**Authors:** Kimberley G Jacobs, John Kugler, Jeffrey Chi, Elizabeth Stuart, Sylvia Bereknyei Merrell, Caroline Rassbach

**Affiliations:** 1 Cardiology, Cincinnati Children's Hospital, Cincinnati, USA; 2 Internal Medicine, Stanford University School of Medicine, Palo Alto, USA; 3 Internal Medicine, Stanford University School of Medicine, Stanford, USA; 4 Pediatrics, Stanford University School of Medicine, Lucile Packard Children's Hospital, Palo Alto, USA; 5 Surgery, Stanford University School of Medicine, Palo Alto, USA

**Keywords:** fourth-year medical students, self-efficacy, capstone courses, boot camp, transition to internship

## Abstract

Background

Capstone, or bootcamp, courses have been shown to increase the knowledge, skills, and self-efficacy of students prior to starting intern year and have been recommended by the Alliance for Clinical Education (ACE) to be incorporated into the fourth-year medical school curricula. However, a paucity of research exists regarding the exploration of the student perspective on critical curricular content and teaching strategies in a capstone course. Self-efficacy, one’s subjective task-specific judgment of capability, has served in the literature as a framework for capstone outcomes and is derived from four sources of experiences: practice, observation of others, feedback, and one’s emotional reaction to difficult situations. Utilizing this framework, we aimed to evaluate the impact of our capstone curriculum on students’ self-efficacy and to identify critical curricular content and teaching strategies that affected students’ self-efficacy and their transition into residency.

Methods

We designed a mixed methods study of our institution’s capstone course in May 2019. Students were invited to participate in the retrospective pre- and post- self-efficacy survey and focus group immediately after the capstone and in semi-structured interviews four months after they began the intern year. Themes were identified via qualitative analysis using inductive coding to allow participants’ voices to guide code development and deductive analysis using codes derived from the self-efficacy framework.

Results

Nine enrolled students participated in the study (surveys n=8, focus group n=7, follow-up interview n=6). Students reported the capstone was a very valuable educational experience (median 4.5 [interquartile range, or IQR 4-5]), increased their preparedness for intern year (median 5 [IQR 4.25-5]) and increased self-efficacy in multiple domains. Qualitative analysis revealed the critical curricular elements that most impacted students’ self-efficacy were practical and communication skills to which students previously had limited exposure, in particular managing acute clinical needs, overnight cross-cover pages, inpatient pharmacology, daily intern communication (handoffs, consults, consenting), and end-of-life communication (goals of care, code status, pronouncing death). While all four sources contributed to self-efficacy, students reported that instructor and peer feedback were fundamental to providing context and substance to their performance. Students preferred practice-based learning via high-fidelity simulation and small groups for familiar tasks (daily intern communication, overnight pages, pharmacology) and observation of peers for new tasks (end-of-life communication and acute clinical deterioration).

Conclusions

This is the first study describing students’ perspectives on critical curricular content and teaching strategies for a capstone course derived from qualitative analysis. Practical and communication skills with previously limited clerkship exposure and task-specific learning strategies increased the students’ self-efficacy. Constructive feedback provided an important source of self-efficacy for all tasks, augmenting the benefits of practice and observation. This data provides preliminary groundwork for future research as multi-institutional studies are necessary to better understand students’ needs around the curriculum to address residency transition.

## Introduction

The transition from medical student to intern is described as a steep learning curve, with some senior medical educators perceiving new residents as inadequately prepared to be interns [1,2]. The new responsibilities and independence contribute to interns’ burnout [3], stress [4], depression [5], and, in some cases, even regret in career choice [4]. Capstone courses, also referred to as boot camps or intern preparatory courses, were developed to address the abruptness and negative impacts of this transition by improving student preparation for their upcoming roles as physicians [6,7]. These courses are recommended by the Alliance for Clinical Education (ACE) as a standard component of fourth-year medical student curricula [1] prior to graduation and implementation is increasing across the United States [8-10].

Multiple institutions have published the short-term outcomes from their capstone courses demonstrating improved clinical skills, knowledge, and self-efficacy in fourth-year medical students [11-14]. Specifically, capstone courses were identified as one of the most important parts of students’ medical education and resulted in increased student confidence which sustained several months into intern year [12-14]. These findings support the ACE recommendation that capstone courses are important innovations in the final year of medical school curricula. However, significant variability exists between institutions in capstone content and teaching modalities [9-11, 15]. In 2014, the ACE recommended 15 core capstone topics to address potential gaps in medical school education based on clerkship directors’ recommendations [1], but these topics have not yet been validated with student input or outcome measures such as self-efficacy.

Self-efficacy is an important framework in the transition to residency and is defined as one’s judgement of one’s own capability to learn or perform. In this framework, self-efficacy can vary by task, context, and goal [16], and is derived from four sources: mastery experiences (i.e. practice), vicarious experiences (i.e. modeling), verbal persuasion (i.e. constructive feedback), and physiological states (i.e. one’s emotional reaction to a difficult situation) [17]. Success from prior mastery experiences can generalize to a sense of capability in future stressful/difficult situations. Vicarious experiences provide encouragement that if others, particularly peer models, can complete the task, then the learner can as well [18]. Verbal persuasion encourages learners and corrects performance for success. One’s physiological state provides a reflection of one’s perceived competence or anxiety which either encourages or inhibits learning, respectively [17,18]. Despite a poor correlation between a physician’s self-assessment and true competence [19], the strength of a learner’s self-efficacy is believed to affect their coping strategies, activity choices, effort, and persistence [17,20].

The purpose of this study was to (1) evaluate the effect of our capstone curriculum on students’ self-efficacy; (2) identify critical curricular content in a fourth-year medical student capstone course that impacted student self-efficacy; and (3) explore how specific teaching strategies utilized the four sources of self-efficacy to affect students’ self-efficacy.

## Materials and methods

This was a prospective convergent mixed methods cohort study of graduating medical students who participated in Stanford School of Medicine’s capstone course. All medical students who participated in the capstone course in May 2019 were eligible for recruitment. The study was approved by Stanford University’s internal review board and informed consent was obtained from every participant.

Capstone curriculum

The Stanford School of Medicine Capstone curriculum was first developed by the authors JC and JK in 2013 in response to a local needs assessment of medical school and residency leaders at Stanford. The curriculum has been adjusted yearly based on student feedback and currently exists as a one-week multi-modal course consisting of high-fidelity simulations, workshops, small group activities, and supporting didactics. Three simulated patients formed the core of the curriculum with various learning activities incorporated throughout the week enabling students to make clinical choices that impact hospital courses and thereby become active participants in their patients’ care. Students managed medical emergencies and experienced difficult conversations with patients and families through simulations that built upon one another throughout the week. Students signed patients out to each other at the end of each day and were paged overnight by instructors on four to five issues that arose during the night. All simulation experiences, including overnight pages, were followed by debriefing sessions and feedback.

**Figure 1:**
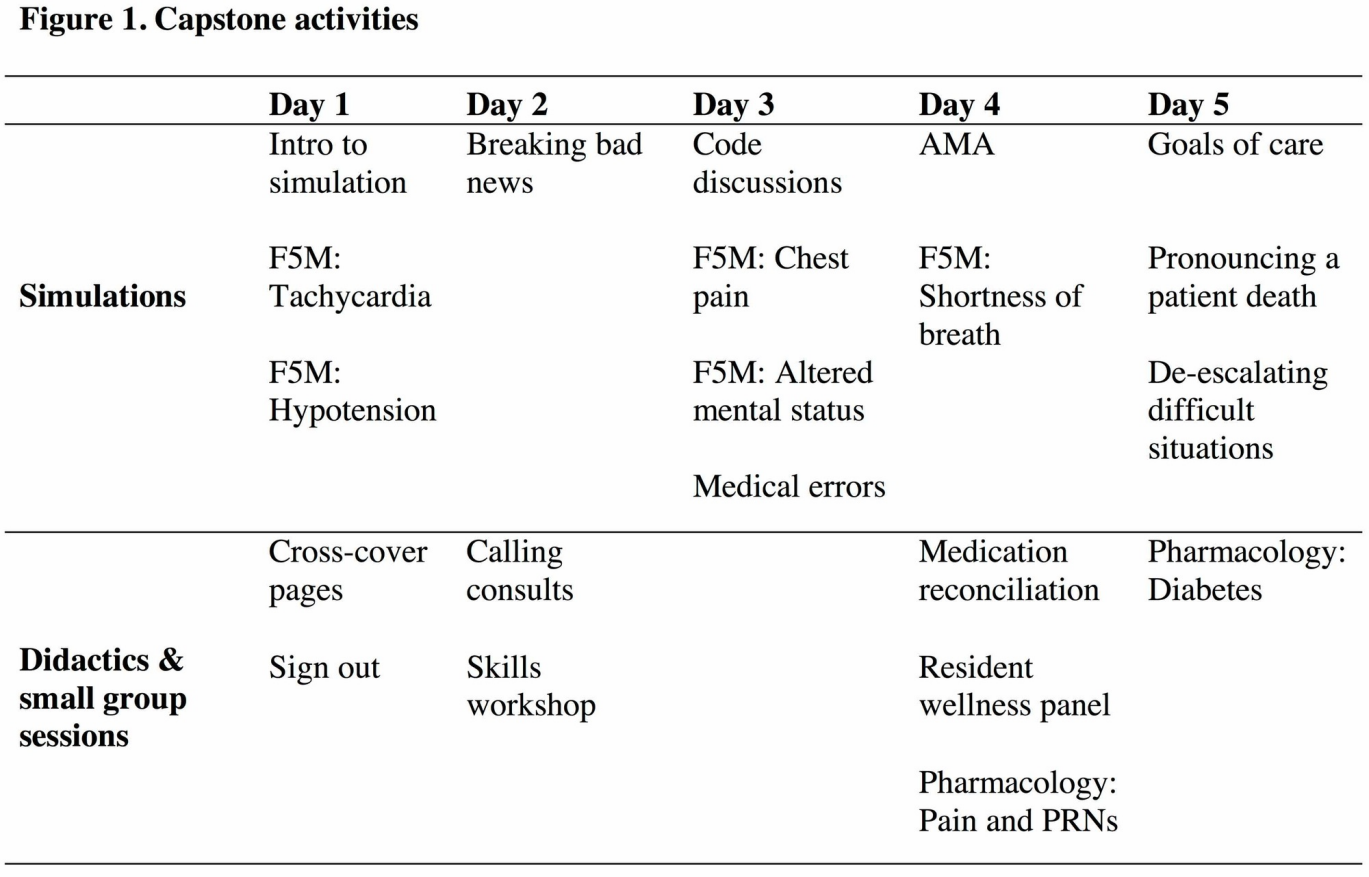
Capstone curricular content Chart displaying the capstone curricular content (order of activities not depicted). Days 1-5 ended with students signing out patients and their events to each other as preparation for the overnight cross-cover call. Days 2-5 began with a one-hour session reviewing pages from the overnight sessions and reviewing sign-out. Skills workshop included stations on IV and Foley placement, suturing, and advanced airways. F5M = First five minutes; AMA = against medical advice; PRN = pro re nata (common medical abbreviation for “as needed”).

The components of the 2019 curriculum addressed eight of the 15 ACE recommended domains [1] including (1) management of common clinical scenarios that may be encountered while providing cross-coverage; (2) basic procedural skills; (3) effective consultation skills; (4) communication at the end of life; (5) obtaining informed consent; (6) effective “sign-out” of patients; (7) recognizing and understanding roles of other members of the healthcare team; (8) stress management (Figure 1).

Data collection

Quantitative Data

We developed a 22-item retrospective pre/post survey to address our first aim of assessing change in student self-efficacy. Self-efficacy survey content and question stems were developed based on published best practices [21,22]. The survey questions were created to assess the impact of the curriculum on students’ self-efficacy in performing tasks within the eight ACE domains addressed in the curriculum. The survey underwent review by experts in medical education research (internal medicine and pediatric clerkship directors and residency leaders) for content validity [21]. We pilot tested the survey and performed cognitive interviewing with five pediatric interns for response process validity [22,23], resulting in minor modifications to the wording of the questions. The final survey included 20 self-efficacy items on a 5-point Likert certainty scale [1=not at all certain; 5=very certain] and two questions assessing overall educational value on a 5-point Likert agreement scale [1=strongly disagree; 5 = strongly agree] (Appendix). The survey was administered via paper on the last day of the course.

Qualitative Data

We addressed the second and third aims of identifying curricular content and teaching strategies that impacted student self-efficacy through a focus group with participants immediately after the course and through semi-structured interviews of the participants four months after they started intern year. The focus group and interview guides were developed based on review of the literature [1,24] and were piloted with the same five interns using cognitive interviewing (see Appendices). The focus group consisted of six questions to explore students’ experience with the capstone course as well as what they identify as critical curricular elements and teaching strategies that they perceive to have impacted their self-efficacy. The semi-structured follow-up interviews mirrored domains in the focus groups and included four questions assessing the content and impact of the course on students’ transition to intern year and opportunities for curricular improvement based on their experience as interns.

Focus groups were conducted by the first author on the last day of the capstone course and recorded using a tablet with the Rev transcription service app (Rev.com Inc., San Francisco, CA). Audio-recordings were transcribed verbatim. The first author verified the transcription quality by replaying audio recordings and editing transcripts. The follow-up interviews were performed via telephone by the first author during September through October 2019, similarly recorded, and transcribed verbatim. Students received lunch on the day of the focus group and a $50 gift card if they participated in the structured interview.

Data analysis

The survey, focus group, and interview data were analyzed independently and interpreted together to determine if findings complemented or diverged [25]. Survey data are reported as median IQR. The pre/post self-efficacy survey was analyzed via Wilcoxon signed-rank test using SPSS v.25 (IBM Corp., Armonk, New York). The level of significance was set at p<0.05. The qualitative data underwent thematic analysis starting with a systematic coding process done both inductively to allow participants’ voices to guide code development and deductively using codes derived from the self-efficacy framework [17,20]. The process was completed based on the three-pass approach described by Hanson et al [26]. The data, including field notes and transcriptions, were reviewed by four authors (KJ, CR, JC, JK) to familiarize each author with the data. Two authors (KJ, CR) independently coded the transcripts, developed a code book (pass one), and then re-analyzed the transcripts for preliminary themes and identification of quotes and excerpts that addressed the study aims. These themes were then discussed among all authors to develop thematic consensus (pass two). Disagreements between the first two coders were arbitrated by JC and JK. Reiterative review of the data resulted in development and re-organization of themes which were agreed upon by all authors (pass three). The interview data underwent a similar coding process with the same codebook and theme development, and provides additional value as part of the explanatory mixed methods study [25].

## Results

Nine students participated in the one-week capstone course and eight agreed to participate and complete the surveys. These students (five male, three female) represented future family medicine, internal medicine, OB-GYN, surgery, and anesthesia residents. Six students attended the last day of the capstone course and participated in the focus group; one additional student who left early agreed to answer focus group questions by e-mail. Six students participated in the telephone follow-up interviews. All data were combined for qualitative analysis.

Effect of capstone curriculum on students’ self-efficacy

Students strongly agreed that the capstone course was a valuable educational experience (median 4.5 [IQR 4-5]) and made them feel more prepared to start intern year (median 5 [IQR 4.25-5]). Student self-efficacy increased for tasks in six out of the eight ACE domains (Table 1). The most pronounced statistically significant improvements were in managing common clinical scenarios, calling consults, communicating at the end-of-life, obtaining informed consent, and signing-out effectively. In contrast to these five tasks, the capstone did not statistically significantly improve students’ self-efficacy in the following three areas: performing procedures, understanding interprofessional relationships, or managing stress.

**Table 1:** Fourth-year medical students’ ratings of self-efficacy before/after the capstone Retrospective pre/post self-efficacy survey on a five-point Likert scale of certainty anchors [1 = not at all certain; 2 = somewhat certain; 3 = fairly certain; 4 = certain; 5 = very certain]. The double lines delineate separation of questions into each of the eight ACE domains. n = 8. *p<0.05. RN: registered nurse; RT: respiratory therapist

ACE Domain	Survey Question	Before Median [IQR]	After Median [IQR	p-value
Management of common clinical scenarios	Start the initial steps of stabilizing a patient?	2 (2-2.75)	3 (3-3)	0.011*
	Call for help and escalate care for an unstable patient?	3 (2-3)	4 (3.25-4)	0.015*
	Communicate effectively in an unstable patient situation?	3 (2-3)	3.5 (3-4)	0.011*
	Prioritize the steps of the work-up for an unstable patient?	2 (2-3)	3 (3-3)	0.020*
Procedural skills	Insert a Foley catheter?	3 (3-4)	4 (3-4)	0.046*
	Bag-mask ventilate a patient?	3.5 (3-4)	4 (3.25-4)	0.157
	Obtain IV access?	3 (2.25-3)	3 (2.25-3)	0.317
Effective consultation skills	Formulate and ask an appropriate consult question?	3 (3-3)	4 (3-4)	0.025*
Communication at the end of life	Break bad news?	3 (2-3)	4 (3.25-4)	0.014*
	Elicit patient and family wishes (i.e. goals of care)?	3 (2-3)	4 (4-4)	0.008*
	Determine a patient's capacity to make medical decisions?	2 [2-3]	3 (3-3.75)	0.020*
	Discuss code status with a patient and a family?	2 (2-2.75)	3.5 (3-4)	0.009*
	Pronounce a patient's death?	1 (1-1.75)	3 (3-3)	0.020*
Obtaining informed consent	Explain the risks, benefits, and alternatives of a procedure to a patient?	2.5 (1.25-3.75)	3 (3-4)	0.063
	Assess a patient's understanding of the risks, benefits, and alternatives?	2.5 (2-3)	3.5 (3-4)	0.023*
Effective “sign-out” of patients	Give sign out to your receiving team?	3 (2-3)	4 (3.25-4)	0.011*
	Develop a contingency plan for each patient as a part of your handoff?	2 (2-3)	3 (3-4)	0.008*
Recognizing and understanding the roles of other members of the healthcare team	Coordinate the roles of multidisciplinary members of your team for patient care?	3 (2-4)	3 (3-4)	0.157
	Identify and utilize the skills of interprofessional team members (ex: PharmD, RN, RT)?	3 (2-4)	4 (3.25-4)	0.063
Stress management	Balance professional responsibilities with personal responsibilities?	3.5 (3-4)	4 (3-4)	0.317

Identification of curricular content that impacted self-efficacy

Five elements of the capstone curriculum were identified as critical content that strongly influenced students’ self-efficacy. These thematic curricular elements aligned with the results of the survey data and found that clinical and communication skills, which students reported minimal exposure to during clerkship years, most strongly influenced self-efficacy (Table 2).

**Table 2:** Curricular elements that students identified as impacting their self-efficacy

Curricular Element Description		
First five-minute Simulations		Students served as first responder to critical pages about a patient and led initial work-up and management. Instructor facilitated debriefings followed and reviewed clinical management and the intern’s role in the first five minutes of an acute clinical situation.
“We really emphasized this week that we don’t have to do everything alone by yourself … Instead, we had an emphasis on teamwork and that we can always call for help was really helpful” (focus group). “You are the provider for these patients… and so knowing what to do in that first five to ten minutes after you’ve called an RRT [rapid response team], really sets you up to succeed” (follow-up interview).		
Overnight cross-cover pages		Students received five pages between 9PM-12AM each night to which they could respond via text or phone call. The following morning students reviewed their responses to pages and analyzed their own and their peers’ replies with input from instructors.
“One of the most important lessons that I learned from the course was that sometimes the best approach is just to watch the patient closely” (focus group). “[The pages] made me realize that the nurses are a critical resource even when in-house because they’ve had eyes on the patient longer than you” (focus group). “I didn’t even know what to expect in terms of getting pages, what they would look like, how do I even respond, what kind of things that I’d even be asked because even as a [sub-intern] I never got pages… so just triaging what’s important and what I should actually go to the patient for … was really helpful going into intern year” (follow-up interview).		
Inpatient pharmacology		Students participated in small group and didactic teaching on medication reconciliation, pain and diabetes management, common “PRN” [pro re nata] requests from nurses. These topics were also incorporated into the overnight paging curriculum.
“In general, pharmacology and […] dosing medication is one of the things that I’m the most nervous about, because I haven’t had to do much of it myself and now I’ll be responsible for prescribing” (focus group). “[I learned] what to consider when you hold different meds or continue different meds and in the context of the patient’s condition and thinking about medications that way. I hadn’t done that a lot as a med student” (follow-up interview).		
End-of-life communication	Students practiced the role of primary communicator during high-fidelity simulations with the standardized patient (SP). These sessions required students to break bad news, discuss code status, discuss goals of care, prevent the SP from leaving against medical advice (AMA), and, ultimately, pronouncing death.	
“The sessions expounded upon important topics that were difficult to sit down and address as a student on clerkships” (focus group).		
Daily intern communication	Students learned communication frameworks for and practiced calling consults, giving handoffs, and consenting patients in small group sessions as part of the care for their three patient arcs. They also participated in a high-fidelity simulation of patient de-escalation with a standardized patient.	
“It felt like consults went a little smoother once I had a more specific structure to what I was doing…it just makes you a little bit more thorough, similar to the handoff practice” (follow-up interview). “I had never consented anyone before. I was able to walk through literally what’s required, what’s not. So that, intern year I wasn’t completely clueless” (follow-up interview).		

The first critical curricular element was the “First Five Minute” simulation series where students were paged to critical clinical scenarios and were responsible for the “first five minutes” of the work-up and management in an acute clinical scenario. These simulations helped students acknowledge their limits, appreciate they don’t need to “know it all,” and learn to use their resources at hand by calling for help. Debriefings focused on feedback and discussion of their role as an intern during acute clinical scenarios which was later identified during structured interviews as one of the most important lessons of the capstone in helping prepare students for intern year.

The second critical curricular element was the overnight paging curriculum. Students noted the angst and difficulty of answering and triaging cross-cover pages. They learned that there are multiple appropriate responses to pages and that the bedside nurse can be a crucial resource for patient information and clinical expertise. This activity particularly led students to reflect on the gravity of being a physician: “Think about how long it took to answer the page. We all knew the patient had a [pulmonary embolism], but none of us wanted to heparinize that patient because, like, is anyone actually going to make the call to give them heparin when they have had a GI bleed three days ago that caused us to have to resuscitate him? That was just a moment of distinguish - from a moment [of being] a med student to a moment [of being] a resident - that, yes, I have the answer, but I also have to deal with the consequences if the answer is wrong. That is a hard thing to navigate (focus group).” Further, students identified this activity at both time points as one of the most effective ways to promote the psychological shift from being a student to being an intern and thereby made them feel more capable of taking on intern year.

The third critical curricular element was prescribing and managing common inpatient medications. Students reported significant anxiety about the impending responsibility of managing medications during the focus group. However, students reported that practice using common resources (ex: UpToDate, Lexicomp) and receiving feedback from their instructors made them feel more capable of prescribing common medications. In follow-up interviews, participants frequently reported these sessions were very helpful for when the clinical scenarios came up during intern year. These sessions helped them think “big picture” about the role of medications in patients’ care. Students provided the feedback that they would have benefited from additional practice with pharmacologic management of common intern issues as part of the capstone curricula (specifically electrolyte supplementation, diuretics, antibiotics, anti-hypertensives, and medication reconciliation).

The fourth critical curricular element was the end-of-life communication series with a standardized patient (SP). These simulations were often students’ first opportunity to speak as the primary communicator to a patient regarding end-of-life care. In focus groups, students stated that the debriefings, which included the SP, gave them the opportunity to reflect on their communication skills and to reinforce and adapt frameworks previously learned in medical school. These debriefings also provided opportunities for students to practice learned communication strategies with one another. During follow-up interviews, students reflected that they seldom had opportunities to be a part of end-of-life communication discussions during intern year and thus relied on this experience during their capstone more greatly than the daily intern communication skills training.

The fifth critical curricular element was the daily intern communication series. Students found communication frameworks (i.e., situation, background, assessment, recommendation [SBAR] for calling consults, structured examples for obtaining consent) helpful for tasks with which they previously had limited experience, and reported that learning these skills made them feel readier for intern year. Some students, during follow-up interviews, reflected that they were able to quickly learn some of these daily intern communication skills on the job during their first few months of intern year, and thus these skills may not have been as essential to teach in the capstone course. In addition to teaching a general framework for consent, students recommended that the capstone course teach the benefits, risks and alternatives of specific procedures commonly performed such as blood transfusions, central lines, and biopsies. Although in the focus group immediately after the course, students did not highlight de-escalation as a critical curricular element; during the follow-up interviews, all students highlighted the de-escalation training as critical and noted that this skill was needed frequently during intern year. The students reflected that they wished the capstone course included more difficult cases to practice de-escalating.

Notably, students commented that several topics during the capstone course were less useful. Specifically, they felt that stress management and time management were best learned with experience during residency. They also described that the capstone was less effective in teaching interprofessional communication skills because nurses were played by physicians. Finally, students reported that the procedures workshops were less useful because procedures are specialty-specific. However, students suggested teaching common procedures such as airway management and suturing as well as teaching the indications, alternatives, and management of common tubes (feeding tubes, urinary catheters, chest tubes) given the practical application across specialties.

Further, students also provided suggestions for four additional topics to add to the course pertaining to the practical aspects of being an intern (Table 3).

**Table 3:** Curricular content suggestions from students

Curricular Element Description		
Electronic medical record (EMR)		Students want to learn efficient use of the EMR, including for common intern tasks including pre-rounding, admitting patients, placing orders, and discharging patients.
“I think it would be neat if there were some way to simulate how to kind of efficiently... I don't want to say workup, but efficiently get the information you need like first vitals, then Meds, then the MAR and results” (follow-up interview).		
Medical notes		Students described a paradigm shift between writing notes as a medical student, with the purpose of impressing instructors, and as an intern, where notes serve as a source of multi-disciplinary communication and a significant portion of intern workflow.
“It would be good to have several examples where attendings tell us what they look for in a history and physical or discharge summary ... If we write an entire paragraph explaining AKI [acute kidney injury], most of the time the attending doesn’t even care about that so, instead, what does the attending or consultant actually care about? ... What do they want? What is helpful? That would make us more efficient” (focus group).		
Discharge planning		Students noted unfamiliarity with the indications for certain discharge dispositions (skilled nursing facilities, long-term acute care facilities, acute rehabilitation), the roles of social workers and case managers in coordinating discharges, and the impact of management decisions on discharge planning (central lines, injectable medications, supplemental oxygen).
“A lot of those things do fall to the intern and we will pick up over time, but I would feel a lot more confident if I knew the processes to getting people out of the hospital which isn’t something we cover in medical school” (focus group).		
Efficiency and wellness	Students desire to learn how current residents optimize their pre-rounding, note-writing, and task-completion efficiency, as well as the physical exhaustion of intern year and their wellness.	
“[It would have been helpful to be taught that] I come in the morning, this is how I organize myself, this is how I get out my notes, this is how much detail I include. It’s nice to do more, but really you’re fine just doing this” (follow-up interview). “Understanding that life still happens in residency … How do you deal with that? How do you seek support?” (follow-up interview).		

Impact of teaching strategy on self-efficacy

Students identified three teaching strategies utilizing the four sources self-efficacy to impact their self-efficacy (Table 4).

**Table 4:** Teaching strategies identified by students that impact self-efficacy

Teaching strategy	
Practice with feedback is the ideal way to learn for familiar tasks	“Just being thrown in and practicing … I feel like having the chance to actually troubleshoot [with feedback] like, ‘Oh, these are the ways I could say this, or state this,’ it’s really helpful” (focus group). “On the wards, sometimes you do practice, but if someone can’t go with you [to observe and provide feedback] then you don’t actually know if you’re doing it right” (focus group).
Peer and instructor modeling with feedback or debriefing is powerful and formative at this learner stage for new tasks	“Seeing what my peers know or honestly, even the lack of knowledge that we had, with the confidence of, ‘Hey I can follow what they’re doing and learn from what they’re doing,’ ‘cause they have the same training as me and if they can do it, I can do it. But, also, they don’t know something and they’re still going to be fine, so I should be fine too. So, I guess modeling [is helpful] from both a learning-from-them and also I can tell myself that it’s-okay-if-I-don’t-know-everything kind of perspective” (follow-up interview).
Learning to recognize one’s emotions of self-doubt, anxiety, and imposter syndrome allows for development of coping strategies which increase one’s ability to perform tasks.	“I used this capstone clerkship to personally advance in the practice of controlling emotions: getting to be in scenarios that could be anxiety-provoking in real life but also in the process of ‘volunteering to go,’ getting used to trying out scenarios when I didn’t have a framework to approach them. It forced me to put aside concerns of ‘looking dumb’ and just focus on the learning. I think the latter is a crucial skill that I will need in residency” (focus group). “I have to quell that voice that says you’re in the way and start to remind myself like at some point there won’t be an attending to hand this or when at some point you will be a chief and someone will be looking to you to hand it over to and so you will have to go through this and deal with it now so what you are ready at that point” (follow-up interview).

The first teaching strategy that students reported as valuable for fostering their self-efficacy was practising common intern skills with their immediate instructor, peer, and SP feedback. Students preferred to learn topics they had some familiarity with through simulations and rapid cycling exercises (hand-offs, consults, medication prescription, and, for some, acute medical management). Students stated that peer feedback and SP feedback added important depth to the debriefing discussions and stimulated self-reflection. They noted that the continuity of instructors created a safe and confidential learning environment, which was important for practice-based learning. They also recognized that instructors optimized the realism of the course material and allowed students to actively participate in the patients’ hospital courses, which positively impacted their “buy-in” and participation, “I feel like following the patient through the week gave us the right amount of background information which made the sign outs and simulations feel more real to me. It’s not just an abstract patient, we took care of the patient yesterday and we did this which led to this today and now we are talking about it (focus group).”

The second teaching strategy that students reported that fostered their self-efficacy was peer and instructor modeling with subsequent participation in feedback debriefings. Witnessing their peers succeed increased their own self-efficacy, particularly for new tasks such as end-of-life care communication. Observing their peers practice and actively participating in the debriefing sessions helped students “find their words” and brainstorm management and communication strategies. Interestingly, students didn’t feel the need to practice every scenario and could learn from modeling and observation.

The third teaching strategy that fostered self-efficacy was learning to recognize and manage emotional reactions. During debriefings, students explored their stress and anxiety related to starting intern year. Students felt “fear[s] of inexperience”, which led them to “paralysis” during simulation. Students felt both relief and encouragement from discussing these fears and felt comradery in hearing about their peers’ similar reactions.

## Discussion

We present the results of a mixed methods study designed to address three aims including the effect of our capstone course on student self-efficacy, the identification of critical curricular content impacting self-efficacy, and exploration of teaching strategies that increased self-efficacy. To our knowledge, this is the first study utilizing qualitative data from students to evaluate a general capstone curriculum through a lens of self-efficacy.

First, our data demonstrated that students highly valued the capstone curriculum and reported increased self-efficacy after the course. Students’ self-efficacy was most increased for practical intern skills including communication skills (handoffs, consults, and consents; end-of-life communication) and clinical skills (acute clinical management; triaging and answering overnight pages; pharmacology). This finding aligns with the work of prior groups that have reported the most important curricular content for fourth-year medical students focuses on practical intern skills to which students had limited exposure during their clerkship years [1,13,15,27,28]. Similar to the findings reported from surgery-specific capstone courses, learning activities related to answering pages, managing acute clinical deterioration, and prescribing common medications were felt to be particularly valuable and led to the greatest growth in preparing students for intern year [9,15,27,29].

Second, we highlighted five elements of critical curricular content that increased students’ self-efficacy going into intern year and remained critical several months into intern year. At both time points, students recognized the “first five minutes” and overnight paging curricula as highly relevant. However, we noted a few distinctions between student needs prior to starting intern year and partway through intern year. Specifically, the impact of practice with pharmacology cases was noted to be particularly helpful after starting intern year. One potential reason for this is the frequency this task comes up during intern year to which medical students may not be as intimately familiar as they place orders less frequently. This finding is in contrast to the perspective held by program directors that teaching skills in capstone curricula are more important than teaching knowledge [2]. Furthermore, students highlighted in the structured interviews that de-escalation and end-of-life communication simulations with specific feedback were also particularly valuable as opportunities for feedback by attendings and peers during intern year was less common. These findings align with previous qualitative work in which interns reported difficult communications as one among their greatest challenges [29].

Third, we found that while students learned different skills from all four sources of self-efficacy [17], honest and explicit feedback (verbal persuasion) was the foundation upon which the impact of other sources of self-efficacy, namely practice (mastery experiences) and modeling (vicarious experiences), were rooted and amplified. The process of self-reflection and requesting feedback stimulated students’ development of self-efficacy, a notable finding as previous research showed that residency program directors believe self-reflection and soliciting feedback are expected but often missing skills for incoming interns [2]. Students in our capstone course were highly reflective and frequently sought feedback, which may be due to a combination of students choosing this experience as well as the safe and confidential learning environment noted by students that allowed them to express vulnerability. Students preferred practice-based learning via high-fidelity simulation and small groups for familiar tasks (daily intern communication, overnight pages, pharmacology), and preferred observation of peers for new tasks (end-of-life communication, acute clinical simulations). If their peers could perform the tasks, students felt they could probably also perform the task and thereby observing their peer provided a source of self-efficacy even despite a lack of individual practice. We considered this an important finding as the feasibility of allowing every student to participate in a simulation is limited and dependent on institutional resources.

**Figure 2:**
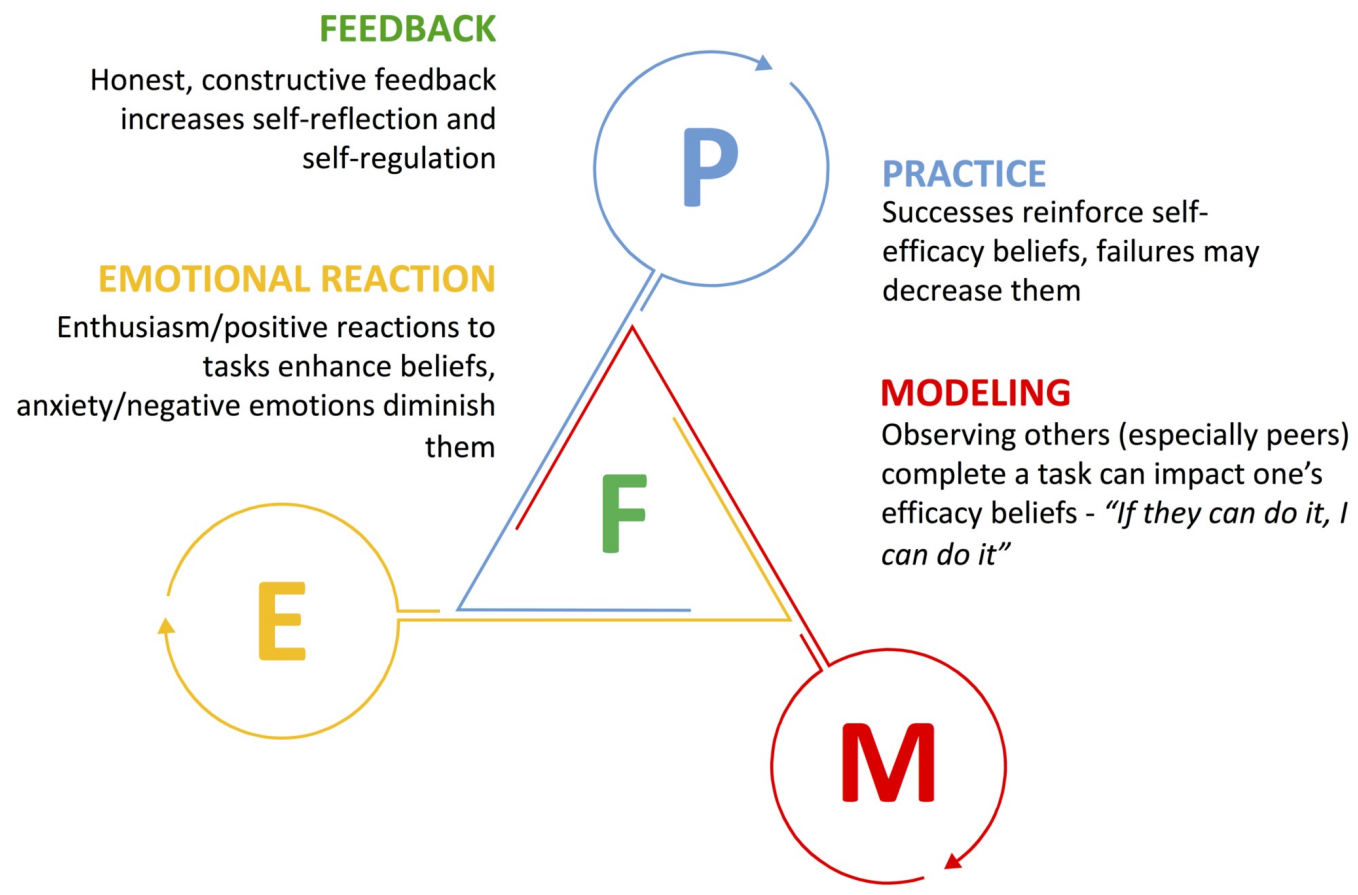
The four sources of self-efficacy Visual model of Bandura’s four sources of self-efficacy incorporating our findings that feedback serves to root and amplify the benefit of practice, modeling, and emotional reactions as sources of self-efficacy.

Though self-efficacy has been used as an outcome measure to study capstone courses via surveys, qualitative research exploring the impact of capstone curricula on medical students’ self-efficacy is limited. Essential to motivation, a learner’s self-efficacy beliefs, or task-specific self-confidence, are postulated to be as important to performance as having the skills and knowledge requisite to the task. In other words, a student needs both “the skill and the will” to succeed [20]. Bandura proposed that students require a strong sense of self-efficacy and resilience to tackle difficult tasks and persevere, which is a relevant concept in the field of graduate medical education which faces increasing levels of burnout [17,20]. Using Bandura’s four sources of self-efficacy and our findings, we recommend that capstone course directors consider incorporating the critical curricular content identified in this study as well as specific feedback and debriefing opportunities to enhance the educational benefit of practice, observation and modeling activities (Figure 2).

Limitations

Our study has several limitations. We had a small number of participants from a single institution. In the initial design, 30-40 students were expected to participate in the capstone course based on average participation rates for the preceding six years. However, due to challenges surrounding course registration during the year of the study, only nine students participated in the course. Despite this limitation, we found that by using a convergent mixed methods study design, the qualitative and quantitative data complemented each other and reflected the participants’ perspectives well. In addition, this was a longitudinal study that followed participants across a span of four months to allow them to experience intern year and to reflect back to describe the impact of this capstone course on their transition to intern year. This longitudinal approach also provided the opportunity for participants to make suggestions for how the course could be improved from the perspective of interns [30]. The capstone course is not a required clerkship at Stanford, likely resulting in a degree of selection bias of students who chose to participate in the course. There also may have been a social desirability bias in answering the focus group and interview questions as the first author who performed the focus group and interviews was a resident who also helped facilitate the capstone course. The capstone course was not graded and the first author had no supervisory role within the school of medicine; thus, we felt that having this author lead the focus group and interviews would allow for a richer conversation as students were familiar with her as a near-peer mentor within the course. Finally, our curriculum and self-efficacy survey only addressed eight out of the 15 ACE domains. In the future, we aim to incorporate the other seven domains into our curriculum to better adhere to the ACE recommendations as well as the topics recommended by our participants.

Despite these limitations, our study aligns with and extends the work of other researchers by contributing a longitudinal qualitative approach representing students’ perspectives to identify critical curricular content and teaching strategies that increased students’ self-efficacy in preparing for internship. Future research should consider inclusion of larger sample sizes to better represent the students' perspectives, longer longitudinal follow-up with reflections on the capstone experience at the end of the intern year, and objective assessments of knowledge and skill retention by supervising residents and attendings to understand the short- and long-term outcomes of capstone courses. 

## Conclusions

We found that a capstone course that includes many of the ACE's recommended core topics improves the self-efficacy of fourth-year medical students as they transition to residency. We also found that students valued practical skills sessions that fill exposure gaps from their clerkship years that address core aspects of daily life as an intern. Students valued learning communication skills regardless of which specialty they were entering and found that their capstone learning in communication carried into the first few months of intern year. Students also viewed honest, constructive feedback as an essential element for professional growth. Future considerations for capstone courses should include aligning courses with the ACE curricular content areas deemed to be most important by learners, adding curricular content on the areas requested by students, and optimizing teaching strategies that align with the sources of self-efficacy.

## Appendices

**Table 5:** Retrospective pre/post bootcamp survey

Not at all certain: Would need an expert to tell me exactly what to do (1)	Somewhat certain: Would need or prefer to have an expert working by my side (2)	Fairly certain: Would want an expert to provide targeted advice or serve as a consultant ( 3)	Certain: Would not need an expert, I can perform on my own (4)	Very certain: Would not need an expert, I can teach to others (5)

	Before the course					After the course				
Start the initial steps of stabilizing a patient?	1	2	3	4	5	1	2	3	4	5
Call for help and escalate care for an unstable patient?	1	2	3	4	5	1	2	3	4	5
Communicate effectively in an unstable patient situation?	1	2	3	4	5	1	2	3	4	5
Prioritize the steps of the work-up for an unstable patient?	1	2	3	4	5	1	2	3	4	5
Perform a lumbar puncture?	1	2	3	4	5	1	2	3	4	5
Insert a Foley catheter?	1	2	3	4	5	1	2	3	4	5
Bag-mask ventilate a patient?	1	2	3	4	5	1	2	3	4	5
Obtain IV access?	1	2	3	4	5	1	2	3	4	5
Formulate an appropriate consult question?	1	2	3	4	5	1	2	3	4	5
Break bad news?	1	2	3	4	5	1	2	3	4	5
Elicit patient and family wishes (i.e. goals of care)?	1	2	3	4	5	1	2	3	4	5
Determine a patient's capacity to make medical decisions?	1	2	3	4	5	1	2	3	4	5
Discuss code status with a patient and a family?	1	2	3	4	5	1	2	3	4	5
Pronounce a patient's death?	1	2	3	4	5	1	2	3	4	5
Explain the risks, benefits, and alternatives of a procedure to a patient?	1	2	3	4	5	1	2	3	4	5
Assess a patient's understanding of the risks, benefits, and alternatives?	1	2	3	4	5	1	2	3	4	5
Give sign out to your receiving team?	1	2	3	4	5	1	2	3	4	5
Develop a contingency plan for each patient as a part of your handoff?	1	2	3	4	5	1	2	3	4	5
Coordinate the roles of multidisciplinary members of your team for patient care?	1	2	3	4	5	1	2	3	4	5
Identify and utilize the skills of interprofessional team members (eg: PharmD, RN, RT)?	1	2	3	4	5	1	2	3	4	5
Balance professional responsibilities with personal responsibilities?	1	2	3	4	5	1	2	3	4	5

Focus Group Questions

1. Many students find capstone courses really useful as they transition to being an intern. What were some of the highlights of the capstone course?

     a.     Second question: How has the capstone course impacted your feelings about starting intern year?

     b.     Prompt: What did you learn from the course?

     c.     Prompt: Tell me more about that, what made it useful?

2.     If you could help the course directors improve the capstone course, what suggestions would you give?

     a.     Prompt: What could be improved or changed?

     b.     Prompt: What sessions could be removed or added?

3.     Which capstone topics/sessions were helpful, if at all, in making you feel more capable to start intern year?

     a.     Prompt: Tell me more about that, what made it helpful?

The following questions aim to understand how specific capstone curriculum sessions affect one’s self-efficacy. Self-efficacy is your own judgment of your capability to learn or perform specific tasks. This week’s course schedule can be found in front of you. Please take a look at the schedule and self-efficacy visual in front of you. As you can see on the visual, there are four ways through which you can feel more certain that you can do something (i.e. increase your self-efficacy): by actually doing them (practice), by seeing them done well by others (modeling), by getting effective feedback, and by controlling one’s emotions so your stress response decreases.

1.     Please think about skills/tasks you will be performing as an intern. Which sessions from this week were useful for learning these skills/tasks? Which was the most useful and why?

     a.     Prompt: Attempt to push students to explain what elements (whether it be a skill or a learning strategy or a knowledge tidbit) was important to learning the topic.

2.     Referencing the diagram, how did the teaching strategy make you feel more certain you could (insert topic here)?

3.     Now taking a step back, if you could create an ideal teaching strategy/session to learn this topic, what would it be?

     b.     Prompt: What about this teaching strategy would make you feel more certain you could (insert topic here)?

Semi-structured follow-up interviews

1.     Tell me what you remember the most about the capstone curriculum.

2.     Which aspects of our capstone program were useful to your transition from a medical student to an intern?

3.     What challenges have you encountered during intern year that could have been addressed in the capstone curriculum?

     a.     Prompt: In what ways did you feel unprepared for intern year?

     b.     Prompt: Based on what you just shared, what suggestions do you have to improve the capstone course to better prepare fourth-year medical students for intern year?

4.     Which capstone sessions were really important and relevant to you being a successful intern during your first few months and why?

    a.     Prompt: For example, did the sessions that made you practice skills help the most? Why? How about sessions that featured modeling/watching your peers succeed at tasks, sessions that included feedback, or sessions that made you stressed? 

5.     Do you have any other comments or feedback about the capstone course?
